# Genetic estimates and genome-wide association studies of antibody response in Tanzanian dairy cattle

**DOI:** 10.3389/fgene.2025.1497355

**Published:** 2025-04-24

**Authors:** Luis E. Hernandez-Castro, Elizabeth Anne Jessie Cook, Oswald Matika, Isaac Joseph Mengele, Shabani Kiyabo Motto, Shedrack Festo Bwatota, Bibiana Zirra-Shallangwa, Ricardo Pong-Wong, James Prendergast, Raphael Mrode, Philip G. Toye, Daniel Mushumbusi Komwihangilo, Eliamoni Lyatuu, Benedict E. Karani, Getrude Nangekhe, Ally Okeyo Mwai, Gabriel Mkilema Shirima, Barend Mark de Clare Bronsvoort

**Affiliations:** ^1^ Centre for Tropical Livestock Genetics and Health (CTLGH), Roslin Institute, University of Edinburgh, Roslin, United Kingdom; ^2^ The Roslin Institute and The Royal (Dick) School of Veterinary Studies, The University of Edinburgh, Edinburgh, United Kingdom; ^3^ International Livestock Research Institute (ILRI), Nairobi, Kenya; ^4^ Centre for Tropical Livestock Genetics and Health (CTLGH), ILRI Kenya, Nairobi, Kenya; ^5^ Department of Global Health and Bio-Medical Sciences, School of Life Science and Bioengineering, The Nelson Mandela African Institution of Science and Technology, Arusha, Tanzania; ^6^ Tanzania Veterinary Laboratory Agency, Central Veterinary Laboratory, Dar es Salaam, Tanzania; ^7^ Scotland’s Rural College, Edinburgh, United Kingdom; ^8^ Tanzania Livestock Research Institute (TALIRI), Dodoma, Tanzania; ^9^ International Livestock Research Institute (ILRI), Dar es Salaam, Tanzania

**Keywords:** genetic estimates, GWAS, genomic population characterisation, Tanzanian dairy cattle, serological response traits, health traits, selective breeding and therapeutic target

## Abstract

Identifying the genetic determinants of host defence against infectious pathogens is central to enhancing disease resilience and therapeutic efficacy in livestock. Here, we investigated immune response heritability to important infectious diseases affecting smallholder dairy cattle using variance component analysis. We also conducted genome-wide association studies (GWAS) to identify genetic variants that may help understand the underlying biology of these health traits. By assessing 668,911 single-nucleotide polymorphisms (SNPs) genotyped in 2,045 crossbred cattle sampled from six regions of Tanzania, we identified high levels of interregional admixture and European introgression, which may increase infectious disease susceptibility relative to indigenous breeds. Heritability estimates were low to moderate, ranging from 0.03 (SE ± 0.06) to 0.44 (SE ± 0.07), depending on the health trait. GWAS results revealed several loci associated with seropositivity to the viral diseases Rift Valley fever and bovine viral diarrhoea, the protozoan parasites *Neospora caninum* and *Toxoplasma gondii*, and the bacterial pathogens *Brucella sp, Leptospira* hardjo, and *Coxiella burnetii.* The identified quantitative trait loci mapped to genes involved in immune defence, tumour suppression, neurological processes, and cell exocytosis. We propose that our results provide a basis for future understanding of the cellular pathways contributing to general and taxon-specific infection responses, and for advancing selective breeding and therapeutic target design.

## 1 Introduction

The smallholder dairy cattle sector in Tanzania makes an important contribution to the country’s gross domestic product and is a source of food, income, and employment for Tanzanian households (Swai and Karimuribo, 2011a; Swai and Karimuribo, 2011b; Katjiuongua and Nelgen, 2014). Despite control efforts, animal diseases remain a significant constraint to productivity and profitability in the sector, with an estimated mortality of up to 14% across herds (Swai et al., 2010; Alonso et al., 2015). Cattle pathogens causing abortion (e.g., Rift Valley fever virus (RVFV), bovine viral diarrhoea virus (BVDV) and *Neospora caninum*), and/or zoonoses (e.g., *Brucella abortus*, *Leptospira* hardjo, *Coxiella burnetii*, and *Toxoplasma gondii*) contribute greatly to economic losses, threaten human public health, and are currently circulating at considerable levels across regions of Tanzania (Bwatota et al., 2022; Mengele et al., 2023; Motto et al., 2023; Lankester et al., 2024).

The defence processes (resilience) by which organisms limit pathogen loads (resistance) or the damage caused by given pathogen loads (tolerance) are crucial for the epidemiology of infectious diseases (Råberg et al., 2007; Horns and Hood, 2012; Knap and Doeschl-Wilson, 2020). Changes in the host’s immunological and pathogenesis mechanisms can particularly reduce transmission, and therefore the prevalence, by blocking infections or eliminating pathogens (Ayres and Schneider, 2008; Medzhitov et al., 2012). Natural host genetic variability (e.g., α-thalassaemia, sickle-cell haemoglobin, and G6PDH deficiency) has been shown to protect against deadly *Plasmodium falciparum* infection (Flint et al., 1986; Lelliott et al., 2015; Archer et al., 2018; Kwok et al., 2021). Several genetic variants and genes (e.g., HLA and IFNAR2 genes) linked to host pathophysiological processes have been associated with SARS-CoV-2 susceptibility and COVID-19 severity (Ovsyannikova et al., 2020; Velavan et al., 2021). Recently, a CRISPR-/Cas9-mediated knockout that alters the BVDV binding domain of the CD46 gene showed reduced susceptibility in a cloned calf (Workman et al., 2021; Workman et al., 2023). Therefore, understanding the genetic basis of disease resilience could complement current control strategies and reduce the burden of endemic or emerging infectious diseases (Knap and Doeschl-Wilson, 2020; Hulst et al., 2021).

Given the limited resources and feasibility of deploying disease control options (e.g., vaccination, biosecurity, and contact tracing), health improvement in smallholder dairy cattle via genomic selection (GS) could complement conventional disease control efforts in Tanzania and other low- and middle-income countries (LMICs). For instance, genetic improvement and quantitative trait locus (QTL) identification, mainly for production traits such as milk yield, are already being implemented in smallholder crossbred cattle populations in India (Al Kalaldeh et al., 2021; Al Kalaldeh et al., 2023). GS implementation in smallholder systems such as those in India and East Africa is challenging, but progress has been made in building breeding infrastructure, routine phenotype recording, affordable genotyping, analytical tools, and human capacity (Mrode et al., 2019; Marshall et al., 2019; Ibeagha-Awemu et al., 2019; Mrode et al., 2021; Brown et al., 2016). This work has allowed a better understanding of the genetic architecture of tolerance/resistance to important diseases in African livestock populations [e.g., East Coast fever in cattle and indigenous chicken infectious diseases (Banos et al., 2020; Wragg et al., 2022)].

Host antibody responses due to recent infection or previous exposure are heritable, and the genetic factors influencing these traits have been explored for several human infectious pathogens (Rubicz et al., 2011; Rubicz et al., 2015; Mangino et al., 2017). The heritability of antibody responses to infectious diseases has also been described in several livestock species. For example, Liu et al. (2014) reported heritability estimates of 0.36 (±0.075) and 0.35 (±0.077), respectively, for antibody responses to Newcastle disease and avian influenza virus in poultry. Heritability estimates of 0.10 (±0.05) for antibody responses to *Mycobacterium avium* subsp. *paratuberculosis*, which causes diarrhoea and decreases milk yield, were reported in Danish dairy cattle (Mortensen et al., 2004). Moderate heritability estimates of 0.32 (±0.09) for immune competence after vaccination against *Clostridium tetani* in Angus beef calves suggest an opportunity for immune competency improvement via genetic selection (Hine et al., 2019). Antibody responses to bovine herpesvirus-1 (BoHV-1), which causes latent infectious bovine rhinotracheitis (IBR), had heritability estimates ranging from 0.12 (±0.05) to 0.14 (±0.04) in Irish cattle (Ring et al., 2019). Therefore, including health-related traits (e.g., humoral immune response) in breeding programmes would be beneficial as a long-term tool to reduce the disease burden in livestock.

Understanding the underlying biology of health traits can be achieved by identifying QTL regions associated with disease resistance. Incorporating relevant associated SNPs into custom-designed SNP chip arrays allows higher accuracy in genomic selection breeding programmes, narrows candidate genes for gene expression or editing, and helps develop diagnostic tools and therapeutic agents. Resistance to *Mycobacterium bovis* has been attributed to several regions in the *Bos taurus* genome, such as the loci containing the PTPRT and MYO3B genes (Bermingham et al., 2014). Tolerance to the protozoan parasite *Theileria parva* (East Coast fever—ECF) in cattle has been linked to a locus spanning a paralogue of the FAF1 gene. In field trials, 100% of animals with a tolerance allele survived *T. parva* infection, although this SNP variant explained approximately 31.9% of the total phenotypic variance (Wragg et al., 2022). The knowledge and identification of putative candidate genes have allowed the editing of bovine genomes to express resistance to pathogens such as *M. bovis* or BVDV (Workman et al., 2021; Workman et al., 2023; Wu et al., 2015). In complex trait studies, such as immune response traits, several genes may contribute small effects in parallel, and therefore, the identification of these underlying mechanisms may be challenging at any given time during the immune response process (e.g., activation of the innate and acquired immune response) (Siwek et al., 2015; Gratten and Visscher, 2016; Solovieff et al., 2013; Leach et al., 2010).

The current study quantifies variation in antibody responses to zoonotic and reproductive infectious diseases and estimates their genetic parameters in highly admixed smallholder dairy cattle populations in Tanzania. We investigated the presence or absence of population structure in our data, computed estimates of heritability for the traits under study, and conducted GWAS using a 668,911 SNP chip array in 2,045 crossbred smallholder dairy cattle. The underlying genetic architecture revealed using GWAS allows the identification of potential QTL regions of importance in livestock health and breed improvement programmes. We discuss possible cellular pathways contributing to general and taxon-specific infection responses and the underlying biology of health traits.

## 2 Results

### 2.1 Genotyping and serological traits of Tanzanian smallholder cattle

**FIGURE 1:**
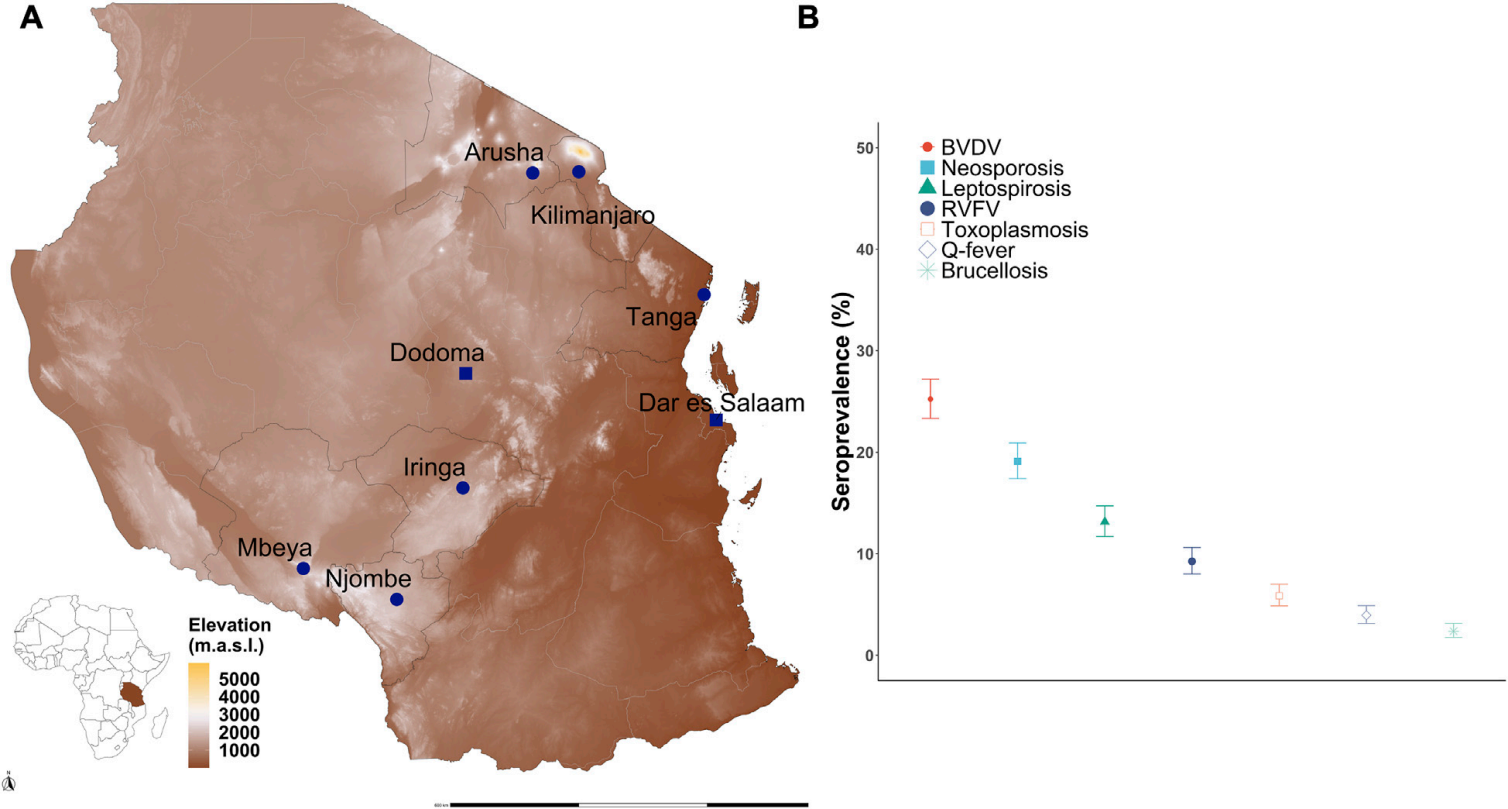
Seroprevalence of reproductive and zoonotic diseases in smallholder dairy cattle in Tanzania. **(A)** Geographic location of the six regions sampled in Tanzania: Arusha, Kilimanjaro, Tanga, Iringa, Mbeya, and Njombe. **(B)** Seroprevalence to seven pathogens among smallholder dairy cattle is variable, with the highest seropositivity to BVDV, neosporosis, and leptospirosis. Source map: www.usgs.gov/centers/eros/science/usgs-eros-archive-digital-elevation-global-multi-resolution-terrain-elevation.

A total of 2,045 crossbred cattle were sampled from six regions that are important for dairy production in Tanzania (Figure 1A) and tested for antibodies to seven pathogens. The animals were initially genotyped with a mid-density (100K) array, and then, the genotypes were imputed to a high-density (∼600K) array (see *Methods*). Thirty-four animals were removed as they failed the quality control (QC) measures from the imputation pipeline due to high levels of missing SNPs. A further 34 animals with high levels of relatedness (>0.25) to another animal based on the KING-robust estimator were removed (that is, one animal from each relatedness comparison pair was removed) (Manichaikul et al., 2010). The call rate (<90%) and minor allele frequency (<0.01) thresholds removed 17,132 of the imputed SNPs. A total of 668,911 SNPs remained after imputation and QC measures for subsequent analyses (see S1 Table for SNP marker density per chromosome). The final dataset contained 1,977 cattle, of which 25.2% were seropositive for BVDV, 19.1% for *N. caninum*, 13.2% for *Leptospira* hardjo, 9.3% for RVFV, 5.9% for *T. gondii*, 3.9% for *Coxiella burnetii*, and 2.4% for *B. abortus* (Figure 1B).

### 2.2 Population structure and ancestry

**FIGURE 2:**
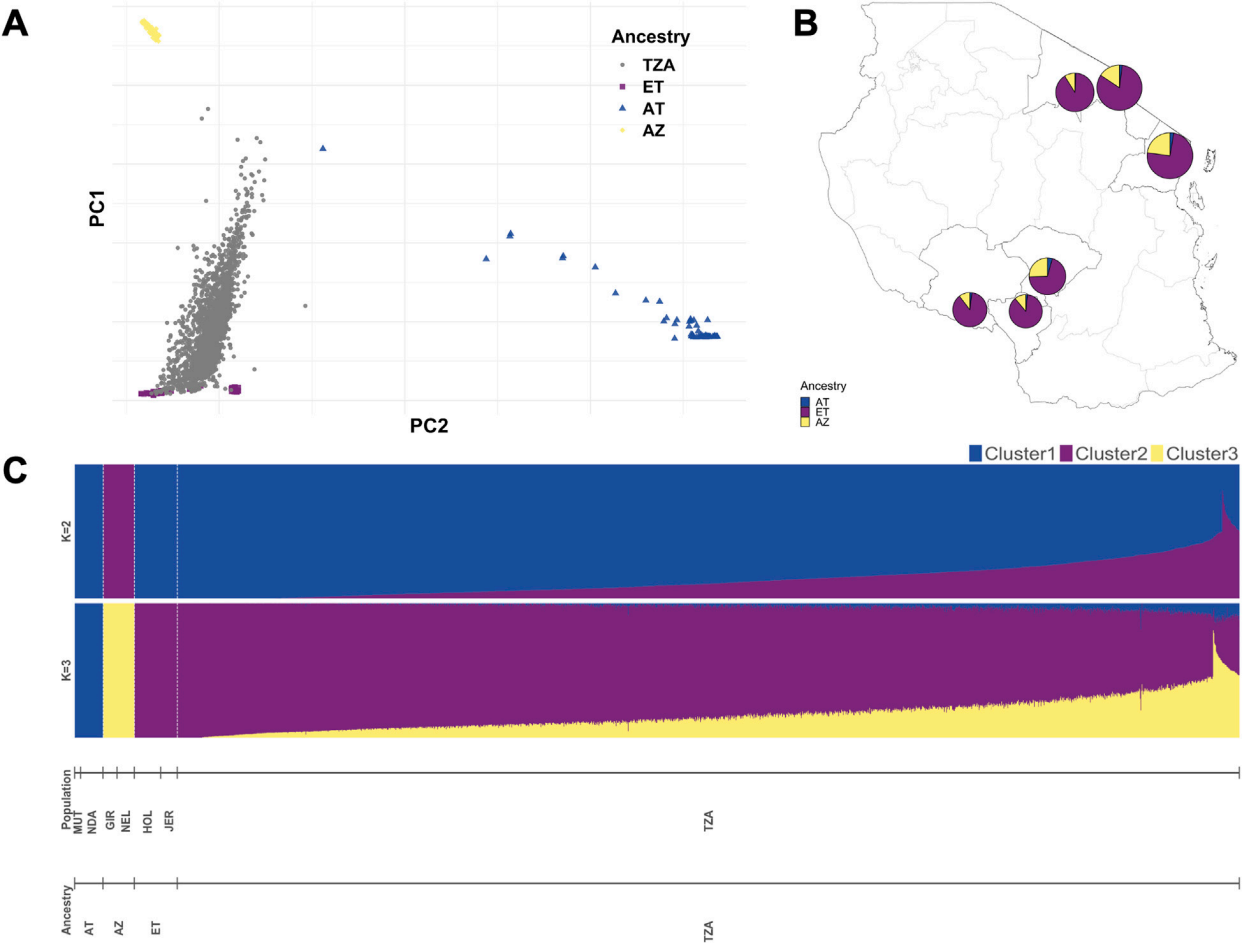
Population structure and global ancestry estimates among smallholder dairy cattle farms in Tanzania, Africa. (A) Scatterplot showing Tanzanian cattle samples (grey dots) within reference populations (European taurine (ET): Holstein and Jersey, African taurine (AT): N’Dama and Muturu, Asian zebu (AZ): Gir and Nelore). **(B)** Pie charts showing ET, AT, and AZ ancestry of Tanzanian cattle in the six sampled regions. **(C)** Supervised admixture analysis of the population using ADMIXTURE software.

We investigated the population stratification and structure among the selected crossbred Tanzanian cattle (TZA) and their genomic context by incorporating purebred data as a reference, after which we performed principal component analysis (PCA) and admixture analysis. The reference cattle population included 99 European cattle (ET) from Holstein and Jersey breeds, 59 African taurine (AT) composed of N’Dama and Muturu, and 65 Asian zebu (AZ) from Gir and Nelore populations (Wragg et al., 2022) (Figure 2A).

First, we visualised the first two principal components (PCs) from the PCA analysis, in which we could identify our reference purebreds. The first and second PCs explained 47.2% and 10.9% of the total genomic variation, respectively (Supplementary Figure S1). We identified five clusters in the PCA scatterplot, where the Tanzanian samples were grouped across ET, where the Holstein and Jersey samples were separated into two clusters, and where the AZ samples from the Gir and Nelore breeds were clustered together. The AT samples, which included the N’Dama and Muturu breeds, were further separated from the rest of the population by the PC2 axis.

We used supervised admixture analysis to estimate the proportion of predefined ancestral populations in our Tanzanian samples (Figure 2C). With *K* = 2 predefined ancestral populations, *B. taurus* and *Bos indicus* ancestry is evident at variable levels in Tanzanian cattle. The estimated global ancestry proportions of *B. taurus* and *B. indicus* were 86% and 14% on average, respectively, across all Tanzanian samples. The proportions of African and European taurine ancestry diverged in our Tanzanian cattle when assuming *K* = 3 fixed ancestral populations. Across all sampled regions in Tanzania, animals had on average high levels of European taurine ancestry, followed by Asian zebu, and, at the lowest level, African taurine ancestry (Figure 2B). Unsupervised admixture analysis revealed similar patterns of ancestries at *K* = 2 and *K* = 3 (Supplementary Figure S2), and more than seven clusters (accounting for six reference populations and our Tanzanian population) were likely present in our dataset, as suggested by the cross-validation plot (Supplementary Figure S3).

### 2.3 Heritability estimates of the serological responses

The seroprevalences in the cattle population under study were low, ranging from 2% for *B. abortus* to 25% for BVDV. We observed low-to-moderate heritability estimates of the serological responses to the seven pathogens likely to cause abortion in our cattle study. The heritability estimates (Equations 1–3), when assuming a continuous trait (0/1) and using the observed scale (h^2^_0,1_), ranged from 0.03 (SE ± 0.06) for Q-fever to 0.44 (SE ± 0.07) for BVDV. However, when we linked the observed estimates (0/1) to the underlying scale (h^2^_u_) [calculated with the formula shown by Dempster and Lerner in 1950 (Dempster and Lerner, 1950; Falconer, 1989)], the estimates were moderate to high at 0.14 and 0.93 for Q-fever and *B. abortus*, respectively (Table 1).

**TABLE 1:** Estimated heritability for serological response to six important reproductive and zoonotic infectious diseases in Tanzanian smallholder dairy cattle.

Serological response trait	*p*	h^2^_0,1_	SE	h^2^u
BVDV	0.25	0.44	0.07	0.81
*Neospora caninum*	0.19	0.19	0.07	0.40
*Leptospira* hardjo	0.13	0.29	0.07	0.74
RVFV	0.09	0.06	0.06	0.18
*Toxoplasma gondii*	0.06	0.23	0.07	0.92
*Coxiella burnetii*	0.04	0.03	0.06	0.14
*Brucella abortus*	0.02	0.11	0.06	0.93

For all serological response traits, the seroprevalence (*p*), heritability on the observed scale (h^2^_0,1_) with standard errors (SE), and heritability on the underlying scale (h^2^u) are provided.

### 2.4 Mapping SNP markers associated with serological response traits

**FIGURE 3:**
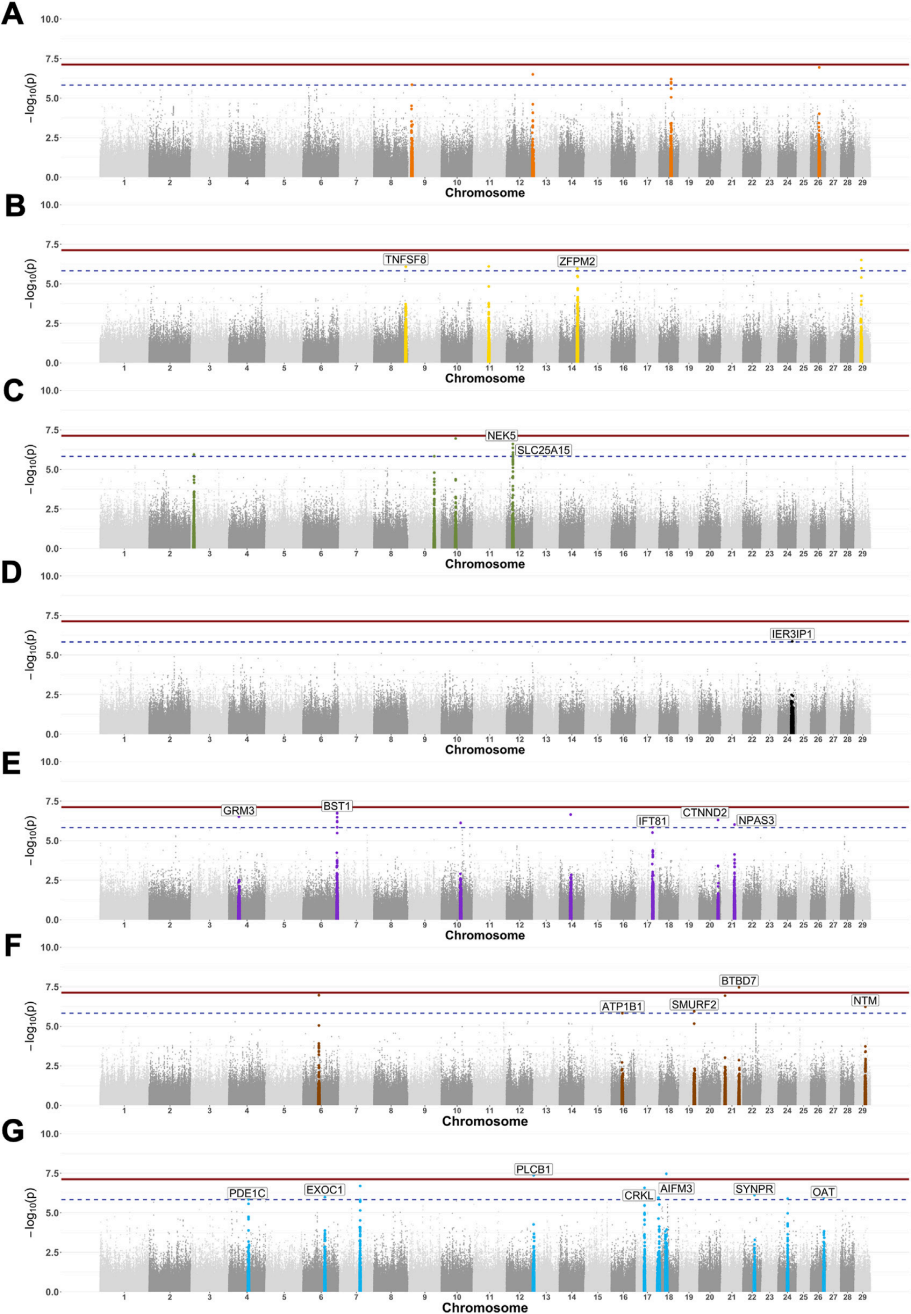
Genome-wide associations for serological response to seven infectious diseases in Tanzanian cattle. Manhattan plots show the SNP markers with the log-10 p-values above 7.13 (continuous line) and 5.83 (dashed line) thresholds. The adjacent 500 SNPs to those significant ones were colour-coded to represent each of the six reproductive/zoonotic diseases: BVDV **(A)**, *Neospora caninum* **(B)**, *Leptospira* hardjo **(C)**, RVFV **(D)**, *Toxoplasma gondii* **(E)**, *Coxiella burnetii* **(F)**, and *Brucella abortus* **(G)**.

Our GWAS results (Figure 3) identified a total of 53 SNP markers with p-values below the suggestive significance threshold ( $p<\;{1.49\;x\;10}^{-6}$ ), and three of these SNP markers crossed the genome-wide significance threshold ( $\left.p<\;{7.47\;x\;10}^{-8}\right)$ across all serological responses to infectious pathogens (Table 2). Thirty SNP markers were mapped to several annotated genes in the *B. taurus* genome (ARS-UCD1.2).

**TABLE 2:** Single-nucleotide polymorphism (SNP) markers in linkage disequilibrium for seven diseases in the Tanzanian smallholder cattle population.

rsID	Chr	Ps	p-value	Gene/Ensembl ID
BVDV				
rs110334415	9	10,632,814	1.41 × 10^−6^	ENSBTAG00000048046
rs136686361	9	10,634,082	1.41 × 10^−6^	ENSBTAG00000056317
rs135177308	12	85,896,180	3.12 × 10^−7^	ENSBTAG00000050193
rs42674898	18	40,128,640	6.15 × 10^−7^	—
rs41614018	18	40,137,391	9.66 × 10^−7^	—
rs134589233	18	40,139,594	1.18 × 10^−6^	—
rs42731467	26	29,327,785	1.13 × 10^−7^	—
*Neospora caninum*				
rs43566009	8	104,087,995	8.37 × 10^−7^	TNFSF8 ENSBTAG00000025782
rs43680152	11	50,640,785	8.02 × 10^−7^	—
rs137229140	14	59,076,463	1.11 × 10^−6^	ZFPM2 ENSBTAG00000001649
rs43059701	29	21,703,572	3.16 × 10^−7^	ENSBTAG00000067594
rs43059696	29	21,705,332	1.04 × 10^−6^	ENSBTAG00000067594
*Leptospira* hardjo				
rs110303769	3	9,566,267	1.14 × 10^−6^	ENSBTAG00000065386
rs135862468	9	82,621,571	1.48 × 10^−6^	—
rs137638789	10	47,042,618	1.10 × 10^−7^	—
rs109573926	12	21,437,462	2.49 × 10^−7^	NEK5 ENSBTAG00000019134
rs134233665	12	21,438,169	4.38 × 10^−7^	NEK5 ENSBTAG00000019134
rs132978619	12	21,541,541	1.20 × 10^−6^	NEK5 ENSBTAG00000019134
rs110000799	12	21,787,162	8.69 × 10^−7^	SLC25A15 ENSBTAG00000011647
RVFV				
rs109147766	24	46,881,767	1.37 × 10^−6^	IER3IP1 ENSBTAG00000032961
*Toxoplasma gondii*				
rs42429616	4	33,738,197	3.02 × 10^−7^	GRM3 ENSBTAG00000018989
rs42732294	6	110,890,410	1.41 × 10^−6^	BST1 ENSBTAG00000006156
rs109950452	6	110,891,679	6.66 × 10^−7^	BST1 ENSBTAG00000006156
rs136210696	6	110,892,348	5.85 × 10^−7^	BST1 ENSBTAG00000006156
rs133473328	6	110,892,915	3.30 × 10^−7^	BST1 ENSBTAG00000006156
rs109159791	6	110,894,364	1.74 × 10^−7^	BST1 ENSBTAG00000006156
rs109667545	6	110,896,924	1.86 × 10^−7^	BST1 ENSBTAG00000006156
rs110528913	10	63,156,501	7.52 × 10^−7^	-
rs41729510	14	37,379,089	2.23 × 10^−7^	ENSBTAG00000032944
rs137128785	17	54,099,612	1.39 × 10^−6^	IFT81 ENSBTAG00000020584
rs109307025	20	62,277,233	4.84 × 10^−7^	CTNND2 ENSBTAG00000017222
rs43031181	21	43,506,933	9.57 × 10^−7^	NPAS3 ENSBTAG00000004462
*Coxiella burnetii*				
rs136314037	6	51,978,781	1.07 × 10^−7^	-
rs41579647	16	36,778,922	1.48 × 10^−6^	ATP1B1 ENSBTAG00000002688
rs41257677	19	48,748,403	1.13 × 10^−6^	SMURF2 ENSBTAG00000019853
rs134852840	21	12,971,743	1.15 × 10^−7^	-
rs132672447*	21	57,923,665	3.41 × 10^−8^	BTBD7 ENSBTAG00000046185
rs137170014	29	34,611,992	5.81 × 10^−7^	NTM ENSBTAG00000010032
*Brucella abortus*				
rs110536633	4	64,608,572	1.42 × 10^−6^	PDE1C ENSBTAG00000002739
rs43008801	6	71,291,921	1.03 × 10^−6^	EXOC1 ENSBTAG00000032637
rs43008805	6	71,294,876	1.03 × 10^−6^	EXOC1 ENSBTAG00000032637
rs43008802	6	71,299,872	1.03 × 10^−6^	EXOC1 ENSBTAG00000032637
rs42880351	7	67,380,512	2.02 × 10^−7^	-
rs42393041*	13	1,495,362	4.30 × 10^−8^	PLCB1 ENSBTAG00000008338
rs109800447	17	27,294,372	2.66 × 10^−7^	-
rs29024118	17	72,302,909	1.09 × 10^−6^	CRKL ENSBTAG00000065402
rs110892009	17	72,312,605	1.21 × 10^−6^	AIFM3 ENSBTAG00000038132
rs134467232*	18	24,663,289	3.42 × 10^−8^	-
rs133958764	22	38,045,884	7.58 × 10^−7^	SYNPR ENSBTAG00000000265
rs109643280	24	31,995,276	1.26 × 10^−6^	-
rs109680241	24	31,996,142	1.26 × 10^−6^	-
rs137397899	24	32,013,683	1.26 × 10^−6^	-
rs132654293	26	44,066,344	1.14 × 10^−6^	OAT ENSBTAG00000006928

The results include reference SNP cluster ID (rsID), chromosome (Chr) base-pair position in the genome (Ps), p-value, and Gene and Ensembl IDs if previously annotated. Asterisks (*) indicate those SNPs above the genome-wide significance threshold.

Seven SNP markers had p-values below the suggestive significance threshold for BVDV and were mapped to unannotated regions on chromosomes 9, 12, 18, and 26 (Figure 3A). Five SNP markers had p-values below the suggestive significance threshold for the *N. caninum* serological response, with one located within the TNFSF8 gene on chromosome 8, a second SNP located within the ZFPM2 gene, and a further three SNPs located within unannotated regions on chromosomes 14 and 29 (Figure 3B). Another seven SNPs with p-values below the suggestive significance threshold were identified for the *Leptospira* hardjo serological response, of which three SNPs were located within the NEK5 gene and one within the SLC25A15 gene, while three SNPs were located within the unannotated regions on chromosomes 3, 9, and 10 (Figure 3C). One SNP marker with p-values below the significance threshold for the RVFV serological response was located within the IER3IP1 gene (Figure 3D). We also identified 12 SNPs with a p-value below the suggestive significance threshold for the *T. gondii* serological response that were located within the GRM3 (one SNP), BST1 (six SNPs), IFT81 (one SNP), CTNND2 (one SNP), and NPAS3 (one SNP) genes on chromosomes 4, 6, 17, 20, and 21, respectively (Figure 3E). One SNP marker with a p-value below the genome-wide significance threshold ( p = 3.41 × 10^−08^) for the *Coxiella burnetii* serological response was located within the BTBD7 gene on chromosome 21. GWAS results also revealed three SNPs with p-values below the suggestive threshold level for the *C. burnetii* serological response, which mapped to the ATP1B1, SMURF2, and NTM genes on chromosomes 16, 19, and 29, respectively (Figure 3F). We also identified 15 SNPs with p-values below the suggestive significance threshold for the *B. abortus* serological response, of which two SNPs had p-values below the genome-wide significance threshold ( p = 4.30 × 10^−08^), with one mapping to the PLCB1 gene on chromosome 13 and another ( p = 3.42 × 10^−08^) mapping to an unannotated region on chromosome 18. The remaining SNPs were in the following genes: one mapping to PDE1C on chromosome 4, three to EXOC1 on chromosome 6, one to CRKL and one to AIFM3 on chromosome 17, one located within SYNPR on chromosome 22, and one within OAT on chromosome 26. The remaining SNPs were mapped to unannotated regions (Figure 3G). QQ plots for each of the seven GWAS are presented in Supplementary Figures S4–10.

To gain further insight into the genetic architecture of these traits, we calculated the proportion of the additive genetic variance explained by the most significant SNPs. The genotype SNP effects were fitted in a linear mixed model to estimate their additive and dominance effects. These effects were used to calculate the allele substitution and the additive variance explained by the SNP. Summary statistics of the SNPs showing the strongest associations are described in detail in Supplementary Table S2. In summary, the proportion of additive variance explained by a given SNP ranged from 0.21% to 1.76%. The allele substitution effects varied from −0.30 to 0.06, and the dominance effects ranged from −0.15 to 0.17. The only two SNPs (rs137229140 and rs42429616) with non-significant additive effects had been mapped to the ZFPM2 and GRM3 genes in the *N. caninum* and *T. gondii* GWAS, respectively.

## 3 Discussion

In this study, we present findings from hard-to-measure phenotypes collected from smallholder dairy cattle herds in the six regions of Tanzania, which were mainly admixed with a high proportion of European taurine ancestry. Our study revealed low-to-high heritability estimates for antibody responses that varied with the pathogens assayed, but were highest for BVDV, *Leptospira* hardjo, and *N. caninum* exposures. GWAS identified several SNP markers with statistically significant associations with the studied traits. The identified loci mapped to *B. taurus* genomic regions, which included annotated genes involved in cell exocytosis pathways and immune response. Our GWAS results are novel and will add to the knowledge of genomic regions associated with cattle immune responses to infectious diseases. Some of the information presented in this study has the potential to enhance our current biological understanding of these traits along with the breeding of cattle for disease resistance.

The history of the domestication of African cattle breeds is complex and marked by multiple admixture events between local African taurine breeds, indicine zebu, and more recently, European (e.g., Jersey, Ayrshire, and Friesian) breeds, resulting in highly diverse populations (Kim et al., 2020). As shown in our population structure and admixture analysis (Figure 2), these Tanzanian smallholder dairy cattle were crosses of European and indicine breeds, comparable to other East African smallholder cattle populations (Mrode et al., 2021; Aliloo et al., 2018), but with higher levels of European introgression. The high percentage of European taurine, followed by indicine zebu, and less African taurine ancestry pattern obtained in the admixture analysis at *K* = 3 can be explained by the sampling of a few African taurine breeds (Figure 2C). The high proportion of *B. taurus* global ancestry (86%) on average in the Tanzanian samples (*K* = 2) may suggest a high proportion of exotic genes. Although our study is limited to investigating global ancestry, we acknowledge that inferring local ancestry for these health traits could be as insightful as it is for production traits such as milk yield (Al Kalaldeh et al., 2023). As purebred data reared under similar conditions were not available, we were not able to investigate genetic differences between breeds and their effect on antibody responses, which have been previously described as a key factor in bovine trypanosomiasis, tuberculosis, and East Coast fever (Wragg et al., 2022; Kim et al., 2020; Callaby et al., 2020). In this admixed Tanzanian cattle population, however, we were able to identify SNPs in linkage disequilibrium with causative mutations responsible for the antibody responses to different infectious diseases.

In this study, the heritability for the seven serological status traits was obtained on the observed scale and converted to the underlying scale. For the observed scale, the binary serological phenotype was assumed to be a continuous variable with normally distributed residuals, and, therefore, the genetic analysis was carried out assuming a standard linear mixed model (LMM). The heritability on the observed scale was transformed to the underlying scale, which assumes that the binary trait is being controlled by an unobserved liability variable, using Robertson’s approximation given in Dempster and Lerner (Dempster and Lerner, 1950; Falconer, 1989). This method of transforming the heritability on the observed scale allows a simple and computationally efficient genetic analysis approach for binary traits while still yielding unbiased estimates of heritability on the underlying scale (Lee et al., 2011). An alternative method to analyse the binary traits in this study would have been to use a generalised linear mixed model (GLMM) with a probit link function to fit the threshold model and account for the unobserved liability variable. While the use of GLMM would be a theoretically more correct approach, given that it allows for better modelling of non-normal data, there are several drawbacks hindering its efficient implementation. GLMM implementations based on penalised quasi-likelihood have been shown to yield biased estimates, and the Laplace approximation and the quadrature methods are less affected by this bias, but at a considerable extra computational cost, especially in relatively complex models (Gbur et al. 2012; Madden and Ojiambo, 2024). GLMM implementations based on the MCMC approach [e.g., Hadfield (2010)] can eliminate the problem of biased estimates, but the computational cost is even greater, therefore restricting its usage in the analysis of relatively small datasets. Hence, our approach of computing the heritability on the underlying scale by transforming the heritability on the observed scale is an appealing alternative, given that it is expected to yield reliable genetic estimates at an acceptable computing cost, while the use of a fast GLMM implementation may not necessarily improve the reliability of the estimates.

The prevalence of the infectious pathogens observed in this study population was variable, with the highest percentages for BVDV, *N. caninum*, *Leptospira* hardjo, and RVFV. These findings partially correspond to the most attributable livestock abortion agents (e.g., RVFV, *N. caninum,* and pestiviruses) found in aetiological surveys in Tanzania (Lankester et al., 2024; Thomas et al., 2022). We observed low estimates of heritability on the observed (0/1) scale when the disease prevalence was the lowest (e.g., 2%); however, the underlying scale that links the observed heritability estimates to the prevalence reflects the true genetic proportion expected for these traits (Dempster and Lerner, 1950; Falconer, 1989; Ojavee et al., 2022) had we used a GLMM appropriate for binary traits.

We identified 53 SNPs with a strong association with serological responses across pathogens—some of which mapped to annotated genes in the *B. taurus* genome (Table 2). The seven SNPs associated with the BVDV serological response mapped to unannotated regions (Figure 3A). BVDV is a highly contagious pathogen with a complex epidemiology that affects dairy cattle herds worldwide by causing persistent infection, poor reproductive performance, and significant economic losses (Scharnböck et al., 2018; Walz et al., 2020). Several BVDV control options exist (e.g., vaccination, biosecurity, and removal of persistently infected animals) (Moennig and Becher, 2018), but they are rarely implemented and/or maintained in LMICs (Richter et al., 2019; Zirra-Shallangwa et al., 2022). With the advances in genome editing, however, it has been possible to breed the first calf with reduced BVDV susceptibility by altering the CD46 gene. BVDV binds to two peptide domains in CD46 to infect cells, and therefore, an altered CD46 molecule appears to limit the viral load in the blood (viraemia) in an edited calf (Workman et al., 2021; Workman et al., 2023). In the RVFV GWAS, one SNP above the suggestive significant threshold mapped to the IER3IP1 gene, mutations of which cause a neurodevelopmental disorder in humans, and it was recently demonstrated to play a fundamental role in B-cell development in mice (Zhong et al., 2023).

The apicomplexan parasites in this study, *T. gondii* and *N. caninum*, are known for causing reproductive problems in cattle and/or have a high zoonotic potential (*T. gondii*) (Reichel et al., 2013). In our *T. gondii* serological response GWAS results, we found that 10 of the 12 associated SNPs were located in the annotated genes, GRM3, BST1, IFT81, CTNND2, and NPAS3 (Table 2). The GRM3 gene encodes proteins that regulate neurotransmitters (e.g., glutamate) and gene mutations have been directly linked to neurological conditions such as schizophrenia (Egan et al., 2004). In addition, GRM3 has also been shown to suppress colon cancer and glioblastoma growth (Yi et al., 2017; Wirsching et al., 2021). The BST1 gene encodes a molecule that facilitates pre-B-cell growth, and it has been involved in autoimmune and neurological diseases in humans (Kaisho et al., 1994; Yokoyama, 2023). The CTNND2 gene has been previously identified in GWAS results from Nelore cattle, where it may be important for growth, meat quality, and milk production (Machado et al., 2022). The rest of the annotated genes are involved in ciliogenesis/spermatogenesis (IFT8) and neurogenesis/schizophrenia disorder (NPAS3) (Michaelson et al., 2017; Qu et al., 2020; van Scheltinga et al., 2010). Investigations into the relationship between toxoplasmosis and the genes involved in neurological processes have only recently been reported (Milne et al., 2020). Several studies have indicated that *T. gondii* infection increases the risk of neurological diseases such as schizophrenia (Carter, 2011; Xiao et al., 2018; Li et al., 2018; Wang et al., 2019). This risk may be somewhat explained by the potential of *T. gondii* to deregulate neurotransmitters, such as glutamate, which is synthesised by astrocytes that are heavily affected during *T. gondii* infection (David et al., 2016). Therefore, it is not entirely unlikely that several of these variants within the GRM3, BST1, and NPAS3 genes may be associated with the host response to *T. gondii* infection, as revealed by our GWAS results.

In the case of *N. caninum,* we identified five SNPs—one mapped to the TNFSF8 gene, a second within the ZFPM2 gene, and three SNPs located in unannotated regions (Figure 3B). The tumour necrosis factor superfamily (TNFSF) genes encode proteins and molecules responsible for host immune defence and tumour suppression (Sonar and Lal, 2015; Ward-Kavanagh et al., 2016). TNFSF8 gene expression plays an important role in the defence of immune cells (e.g., CD4 T cells) against *Mycobacterium tuberculosis* and hepatitis C virus infection (Sallin et al., 2018; Fu et al., 2021). The zinc finger proteins (ZFPM2) are responsible for encoding GATA transcription factors (zinc finger DNA-binding proteins) that control the development of erythrocytes and immune cells such as CD4 T cells (Gao et al., 2015; Lentjes et al., 2016). Although the link between GATA transcription factors and *N. caninum* immune response is unknown, they appear to play an important role in the local immune response to *Pseudomonas aeruginosa* (Shapira et al., 2006).

Several of the SNPs with a significant association with bacterial pathogens (*L.* hardjo, *C. burnetii,* and *B. abortus*) were mapped to the NEK5, SLC25A15, ATP1B1, SMURF2, BTBD7, NMT, PDE1C, EXOC1, PLCB1, CRKL, AIFM3, SYNPR, or OAT genes. In this *B. abortus* GWAS result, three SNPs were located within the EXOC1 gene, which is part of a complex of proteins that regulate cell exocytosis pathways. The EXOC1-encoded protein is part of an 8-molecule complex that binds the cell plasma membrane to endosomal compartments. Several bacterial organisms, such as *Listeria monocytogenes, Staphylococcus aureus*, and *B. abortus* use different exocytic pathways to infect host cells (Ireton et al., 2018; Ireton et al., 2023). After *B. abortus* is phagocytosed by mainly macrophages or dendritic cells, it initially interacts with early endosomes to enter the cell and subsequently replicate within the endoplasmic reticulum (Celli, 2015). Therefore, genetic variations in the EXOC1 region may determine whether *B. abortus* infection will occur or not in host cells. Although the *C. burnetii* serological response association with the NTM gene is unclear, the NTM gene has been identified in association with *B. abortus* infection in wild boar and displaced abomasum disorder in Holstein cattle (Freebern et al., 2020; Fabbri et al., 2022). The association between SNPs located within the BTBD7 and PLCB1 genes and serological response to bacterial pathogens is not clear; however, the BTBD7 gene has been associated with indicators of heat stress in Holstein cattle and is involved in other biological processes such as development and tumour progression (Luo et al., 2022; Liu et al., 2023). The NEK5 and SLC25A15 genes encode proteins involved in mitochondrial function, cell-cycle progression, metabolism and tumourigenesis, but have no clear role in the immune response to bacterial infection (Melo Hanchuk et al., 2015; Chen et al., 2022). *In vitro* human gene expression experiments have shown that the ATPase Na+/K+ transporting subunit β 1 (ATP1B1) protein limits DNA and RNA virus expression and replication by promoting IFN and pro-inflammatory cytokine activation (Cao et al., 2021). Although the SMAD-specific E3 ubiquitin protein ligase 2 (SMURF2) has a primary role in TGF-β signalling pathways (e.g., embryogenesis, and cellular homeostasis), it has recently been shown to affect antiviral signalling pathways (e.g., type 1 IFN signalling and binding of filovirus VP40 matrix proteins) (Pan et al., 2014; Shepley-McTaggart et al., 2021). The PDE1C, CRKL, AIFM3, SYNPR, and OAT genes are involved in developmental and metabolic functions; however, their role in the immune response to bacterial pathogens has not yet been studied.

The SNPs identified as having a strong association with antibody responses to infectious diseases explained a small proportion (0.21%–1.76%) of the additive genetic variance, which is not surprising for complex traits where many genes may be involved in their regulation. However, we were reassured to observe that these SNPs had a significant additive genetic effect, which would allow them to be passed on to the next generation. Importantly, the additive genetic effects in two SNPs that mapped to the ZFPM2 and GRM3 genes were not significant, indicating that dominance effects may be present and play a more important role at this locus. The proportion of genetic variance explained by a given SNP was small, but it is still relevant information that may prove useful in genomic selection and/or gene expression studies to identify genes involved in the antibody response to infectious diseases.

Studying these complex traits with 2,045 sampled animals may be limiting our GWAS, but increasing data collection in livestock systems is often expensive given that health traits are not routinely recorded for breeding purposes. In addition, it is important to note that gathering health trait data from small-scale dairy farms in low-income countries without appropriate cold chain facilities is time-consuming and costly. GWAS results from smallholder systems investigating health traits are scarce, and most studies focus on production performance traits using 4,000–5,000 animals (Al Kalaldeh et al., 2021; Al Kalaldeh et al., 2023; Mrode et al., 2021). Alternately, large-scale commercial cattle GWAS results on production performance traits include between 20,000 and 200,000 animals (Dominguez-Castaño et al., 2024; Jiang et al., 2019). Several GWAS on health traits in commercial cattle populations in developed countries, where phenotypic and genotypic records and financial resources are more likely to be available, could include between 600 and nearly 30,000 animals. To address limited data, flexible Bayesian frameworks have been proposed in crossbred cattle in similar systems while mainly studying production traits such as milk yield (Costilla et al., 2023; Moser et al., 2015). Nevertheless, there is no clear evidence of Bayesian models outperforming GBLUP methods for genomic selection in commercial breeding value evaluation schemes or for health traits in comparable smallholder cattle populations, and GBLUP evaluations are widely accepted in commercial breeding evaluation settings (Mrode et al., 2019; Mrode et al., 2021; Brown et al., 2016).

Despite these limitations, our research provides novel genetic estimates on health that have not yet been reported in smallholder livestock production systems and identifies specific genetic variations linked to the antibody responses to various diseases in admixed African cattle populations. Our findings pinpoint putative regions on the cattle genome that may play a role in immune defence and disease susceptibility. These areas warrant further exploration, such as verification of our findings in other larger similar populations and conducting *in vitro* experiments to study the gene expression or the use of genome editing, as demonstrated in the case of the BVDV-edited calf.

## 4 Materials and methods

### 4.1 Sample collection and study area

Genotypes for the cattle were obtained from the African Dairy Genetics Gains (ADGG) project (https://www.ilri.org/research/projects/african-dairy-genetic-gains), which consisted of 2,045 crossbred Tanzanian cows and bulls sampled from their larger database. ADGG has managed a performance (e.g., milk yield and body weight) and genotype database for the majority of the registered animals, mainly smallholder cattle, from Tanzania and other low- and middle-income countries (LMICs) since 2016. This cattle subset was selected based on the cattle with confirmed presence in both the household and the ADGG database at the time of collection. Households were located in 23 districts in six regions of importance for dairy production in Tanzania (Swai and Karimuribo, 2011a; Swai and Karimuribo, 2011b) (Figure 1).

A cross-sectional survey and sample collection were carried out by our veterinary team, ADGG, and Tanzanian Livestock Research Institute (TALIRI) staff from July 2019 to October 2020. Information on households (e.g., geographic location), animals (e.g., age, sex, and phenotypical features), and herd management (e.g., feeding, reproduction, and health) was recorded electronically using an open data kit (ODK) platform and curated for downstream analysis using R and RStudio (R Core Team. and R, 2022; RStudio Team, 2020).

Blood samples were collected from each animal by jugular venipuncture using plain vacutainer tubes (BD vacutainer^®^, Auckland, New Zealand), centrifuged, and refrigerated until processing in regional laboratories in the study regions of Tanzania. Serum was aliquoted in cryovial tubes and stored at −20°C at the Nelson Mandela African Institution of Science and Technology (NM-AIST) in Arusha, Tanzania.

The animal ethics of the study were reviewed and approved by the International Livestock Research Institute Institutional Animal Care and Use Committee (ILRI-IACUC2018-27), and research approval was granted by the Tanzania Commission for Science and Technology (COSTECH) (ref. 2019-207-NA-2019-95).

### 4.2 Description of the Tanzanian cattle population

On average, cattle in this study were 5 years of age, with 97.2% of them being female breeds. Phenotypic characterisation classified 3.8% as having African indigenous features, whereas 96.2% were identified as crosses between East African Shorthorn Zebu and Ayrshire, Holstein, or Jersey breeds. Management strategies in this population were varied, with the majority of animals placed under an intensive feeding system and having little opportunity to graze freely in open pastures. Reproductive management was carried out through artificial insemination, and a small percentage of farmers reported the use of an owned or rented bull as a mode of reproduction in the herd.

Preventative disease control measures were carried out through vaccination, although they were not routinely implemented, with only 15.2% of farmers reporting using mainly a foot-and-mouth disease vaccine. A total of 23 districts were sampled, with the number of animals in each district varying from 15 in the Iringa Municipal Council to 261 in the Moshi Rural District Council. The herd size, composed mainly of heifers or cows, was variable, with only a few herds being larger than 50 mature females.

### 4.3 Serological health resilience traits

The serological response trait was obtained for each individual animal through testing for the antibody responses to seven pathogens using commercial ELISA kits of BVDV (ID Screen^®^ BVD p80 Antibody Competition, Innovate Diagnostics, France), *N. caninum* (ID Screen^®^ *N. caninum* Competition, Innovate Diagnostics, France), *L.* hardjo (*Leptospira interrogans* subtype Hardjoprajitno and *Leptospira borgpetersenii* subtype Hardjobovis; the Linnodee *Leptospira* Hardjo ELISA Kit™, Linnodee Animal Care, United Kingdom), RVFV (ID Screen^®^ Rift Valley Fever Competition Multi-species, Innovate Diagnostics, France), *T. gondii* (ID Screen^®^ Toxoplasmosis Indirect Multi-species, Innovate Diagnostics, France), *C. burnetii* (Q fever; PrioCHECKIT™ Ruminant Q Fever Ab Plate Kit, Thermo Fisher Scientific, United States), and *B. abortus* (COMPELISA 160 and 400, APHA Scientific, United Kingdom). The manufacturer’s guidelines were followed (see details in (Bwatota et al., 2022; Mengele et al., 2023; Motto et al., 2023; Ryan et al., 2012). All optical density (OD) values were transformed into a binary seropositive seronegative classification based on the manufacturer’s recommended cut-offs.

### 4.4 Genotyping and imputation

Following a method previously applied to East African crossbred dairy cattle (Aliloo et al., 2018), we genotyped our samples using a low-density GeneSeek Genomic Profiler Bovine 100K chip and inferred missing genotypes using a reference cattle population genotyped with a high-density chip. As described in Aliloo et al. (2018), the imputation method uses a crossbred cattle population, mainly from Kenya, Tanzania, Uganda, and Ethiopia in East Africa [see details in Aliloo et al. (2018) Table 1] and a purebred reference (British Friesian, Holstein, Jersey, Guernsey, Nelore, and N'Dama) population (n = 3091), both genotyped using the Illumina BovineHD BeadChip (Illumina, San Diego, CA). This reference population was obtained and curated during the Dairy Genetics East Africa (DGEA) project, which collected performance, genotype, and household data in these East African countries, and was provided by authors Aliloo et al. (2018) solely for imputation. In this procedure, only autosomal SNPs (that is, mitochondrial, unmapped, duplicated map position, and SNPs located in sex chromosomes were removed), with a GC score >15%, a call rate >90%, and a minor allele frequency (MAF) > 0.01, and animals with below 10% missing genotypes were kept for the imputation pipeline. The imputation process involved a pre-phasing stage using the Eagle v2.4.1 positional Burrows–Wheeler transform and hidden Markov model algorithm (Loh et al., 2016), followed by the imputation of genotypes using the Minimac3 v2.0.1 state space reduction algorithm (Das et al., 2016) programme. The dataset after imputation contained 2,011 animals and 670,367 SNPs.

To explore the population structure and global ancestry of our samples, we first merged our samples into a reference population (Wragg et al., 2022) and applied further quality control measures. Both datasets were converted from the TOP to FOR format and aligned using the SNPchiM v3 programme (Nicolazzi et al., 2015) and subsequently merged into a single VCF file using the bcftools v1.3 suite (Li, 2011). The merged data were converted from the VCF to BED format using the Plink v1.90 programme (Chang et al., 2015; Purcell et al., 2007), in which SNPs with an MAF <0.01 (--maf) and a call rate >90% (--geno) and animals with above 10% missing calls (--mind) were removed. Additionally, one animal from a pair was removed for a high degree of relatedness above 0.25 (parent–offspring and full siblings) based on the KING-robust estimator (Manichaikul et al., 2010), which was implemented in the Plink v2.0 programme (Chang et al., 2015; Purcell et al., 2007).

### 4.5 Genomic differentiation and ancestry estimation

We ran PCA and model-based estimation of global ancestry on our Tanzanian dataset and a merged genotype reference dataset to explore the population structure. Our reference population included pure European taurine (ET, n = 99; 63 Holstein, and 36 Jersey), African taurine (AT, n = 59; 47 N’Dama and 12 Muturu), and Asian zebu (AZ, n = 65; 30 Gir and 25 Nelore) cattle genotyped with the Illumina BovineHD BeadChip (Wragg et al., 2022). We also ran a separate PCA with only the Tanzanian samples, with the first five PCs later being used as covariates to control for the population structure in models for genetic parameter estimates and genome-wide association analysis.

Prior to PCA and global ancestry estimation, SNP markers in high linkage disequilibrium (LD) were removed after applying an r-squared threshold >0.2 with another SNP within a 200-SNP window with sliding windows of 10 SNPs at a time. To estimate the level of ET, AT, and AZ ancestry in our Tanzanian cattle, we performed a supervised admixture (Alexander et al., 2009) analysis using a 5-step expectation-maximisation (EM) algorithm. We ran a 10-fold cross-validation with 200 bootstrap resampling to calculate standard errors while assuming *K* = 2 to *K* = 3 fixed ancestries. We compared our supervised analysis with an unsupervised admixture analysis with the same parameter setting but exploring *K* = 2 to *K* = 23 clusters (Supplementary Figure S2). The admixture Q matrix represents the estimated global ancestry proportion of the Tanzanian animals across different genetic clusters. In the Q matrix, the rows show the individuals, and the columns show the clusters identified in the admixture analysis. These clusters were visualised using the R *pophelper* package (Francis, 2017), and the best value of *K* was chosen based on the lowest cross-validation error in the unsupervised analysis.

### 4.6 Genetic parameter estimation

First, we estimated the genetic parameters on the observed scale, and then their heritability ( $h_{0,1}^{2}$ ) was transformed to the heritability on the underlying scale ( $h_{u}^{2}$ ).

The genetic analysis on the observed scale assumes the binary trait (0/1) to be continuous with normally distributed residuals. A single-trait LMM was performed for each trait. The following model was fitted:

y=Xτ+Zu+e, (1)

where y is the vector of observations (serological traits; that is, sero-response to BVDV, *N*. *caninum*, *L.* hardjo, RVFV, *T*. *gondii*, *C. burnetii*, or *B*. *abortus*) modelled on the observed scale (0–1), assuming them as continuous; τ is a vector of fixed effects, u is a vector with the additive genetic effects; X and Z are design matrices associating observations to fixed and random effects, respectively; and e is the vector of residual errors. The fixed effects included in the model were sex (male and female), sample collection month (7 months), districts (23 districts), and herd size (four categories), and the first five PCs obtained from the markers were fitted as covariates. The additive genetic effects were fitted using a genomic relationship matrix (GRM) computed with the genotype information using method 2 of VanRaden (2008). The narrow-sense heritability ( $h_{0,1}^{2}$ ) on the observed scale is estimated as the proportion of phenotypic variance ( $\sigma_{P}^{2}$ ) explained by the additive genetic variance ( $\sigma_{A}^{2}$ ).

$$h_{0,1}^{2}=\frac{\sigma_{A}^{2}}{\sigma_{P}^{2}}.$$ (2)

The heritability on the underlying scale ( $h_{u}^{2}$ ) was calculated by transforming $h_{0,1}^{2}$ based on Robertson’s approximation from Dempster and Lerner (1950) and Falconer (1989).

$$h_{u}^{2}=h_{0,1}^{2}\frac{p\left(1-p\right)}{z^{2}},$$ (3)

where p is the population prevalence and $z^{2}$ is the ordinate of the standardised normal curve corresponding to a probability p . This approximation has been shown to yield unbiased heritability estimates for liability traits (Lee et al., 2011). The model assumes that the underlying scale is a threshold model in which the binary trait is controlled by an unobserved continuous variable (the liability trait), and therefore, the serological status of an individual will be 0 when their liability phenotype is lower than a given threshold and 1 when it is above the threshold (Falconer, 1965).

### 4.7 Genome-wide association analysis

GWAS analyses were conducted using univariate analyses for each of the seven traits using the GEMMA software (Zhou and Stephens, 2012), fitting the same effects included in the LMM described in Equation 1 plus the additive effect of the SNP being tested. The Bonferroni correction was used to account for multiple tests. The SNP effects were declared statistically significant at the suggestive or genome-wide level when p-values were lower than 1.4917 × 10^−6^ and 7.4586 × 10^−8^, respectively. The Manhattan plot (Figure 3) and the QQ plots (Supplementary Figures S4–S10) are shown on the –log10 p-value scale (the suggestive and genome-wide significant thresholds in the –log10 p-value scale are 5.83 and 7.13, respectively).

To get further insight into the genetic architecture of these traits, we investigated the contribution to the overall additive genetic variance for the top SNPs that crossed the genome-wide and suggestive significant thresholds. We fitted an LMM model as described in Equation 1 plus the genotype effect of the SNP in question which, in turn, was used to calculate its additive and dominance effects. These genetic effects were calculated as follows: additive effect, a = (AA – BB)/2; dominance effect, d = AB – [(AA + BB)/2]; and proportion of genetic variance due to SNP = [2 pq ( a + d ( q -– p ))^2^]/V_A_, where the predicted values for each genotype class were defined as AA, AB, and BB, with p and q as the SNP allele frequencies and V_A_ as the total trait additive genetic variance when no SNP effects are included in the model (Equation 1) (Matika et al., 2019; Hadjipavlou et al., 2008; Matika et al., 2016). The predicted values for each genotype class, their (co)variances, and the standard error were obtained using ASReml.

### 4.8 Genome mapping of associated loci

The investigation of the putative genes located with the QTL regions identified for the immune serological response in our Tanzanian cattle was done using the annotated *B. taurus* ARS-UCD1.3 genome in Ensembl.org (https://www.ensembl.org/Bos_taurus/Info/Index?db=core).

## Data Availability

The raw genotype data produced by this project are the property of the Tanzanian government and access to the genotype data is possible if all parties are signatories to the access and benefit sharing documents (prior informed consent and mutually agreed terms) in compliance with The Nagoya Protocol on Access to Genetic Resources and the Fair and Equitable Sharing of Benefits Arising from their Utilization to under the Convention on Biological Diversity. Interested researchers can contact the ADGG project at Support.ADGG@cgiar.org for further information. The serology data can be found at the University of Edinburgh repository at https://datashare.ed.ac.uk/handle/10283/8974.
