# Supplementary material for: Genetic estimates and genome-wide association studies of antibody response in Tanzanian dairy cattle
Source: Front Genet. 2025 Apr 24;16:1497355. doi: 10.3389/fgene.2025.1497355 (PMC12060032; doi:10.3389/fgene.2025.1497355)
Supplement: Supplementary file 1 [file DataSheet1.pdf]

Supplementary Material

1 Supplementary Figures and Tables

1.1 Supplementary Figures

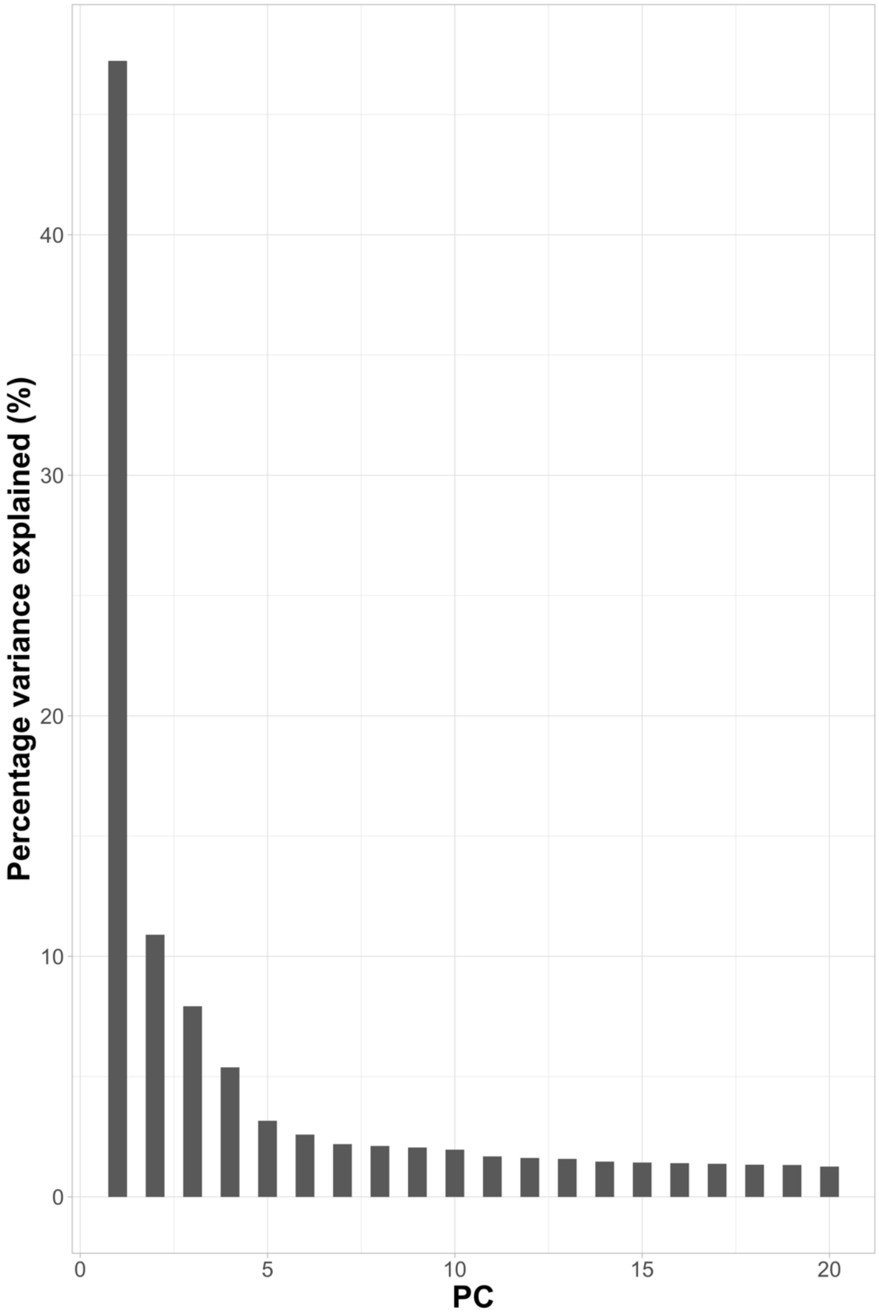

Supplementary Figure 1. Percentage (%) of variance of explained by each of the 20 principal components (PCs) in principal components analysis (PCA) carried out in 1977 Tanzanian animals and 223 reference samples. Bars showed the first and second PCs explaining 47.2% and 10.9% of the total variation.

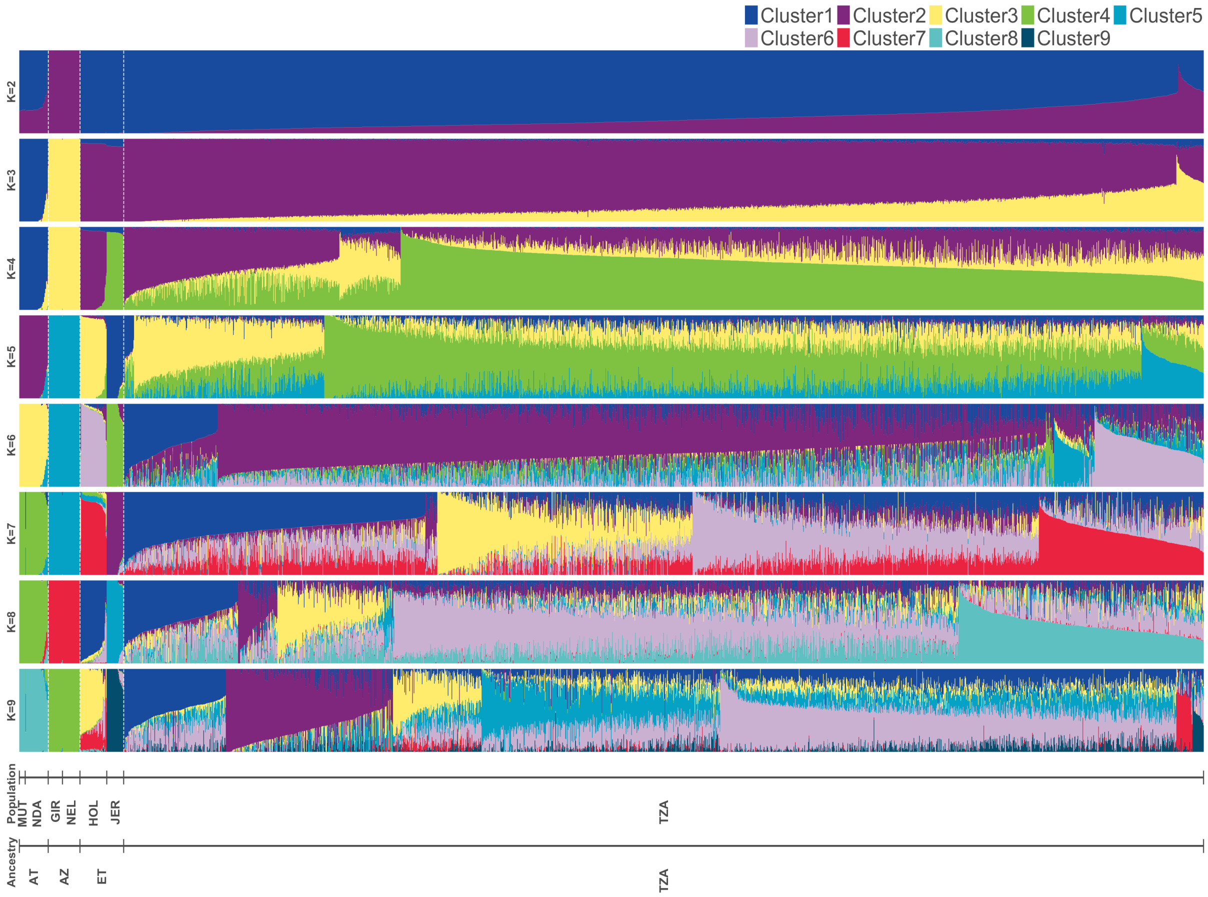

Supplementary Figure 2. Unsupervised admixture bar plot of Tanzanian cattle ancestries assuming *K* = 9 ancestral populations. Bars are split in *K* colours comparable genomic membership to a given ancestral population.

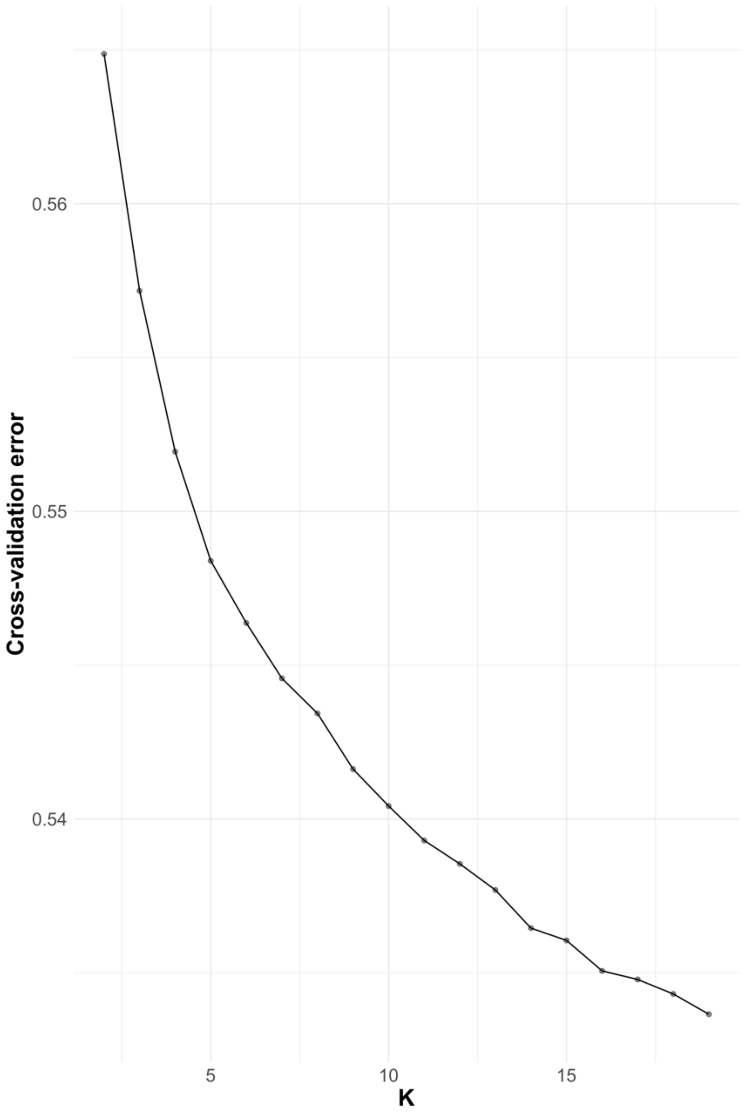

Supplementary Figure 3. Unsupervised admixture analysis cross-validation error plot. Dots-line shows the cross-validation error for a given value of *K*, 2 to 23.

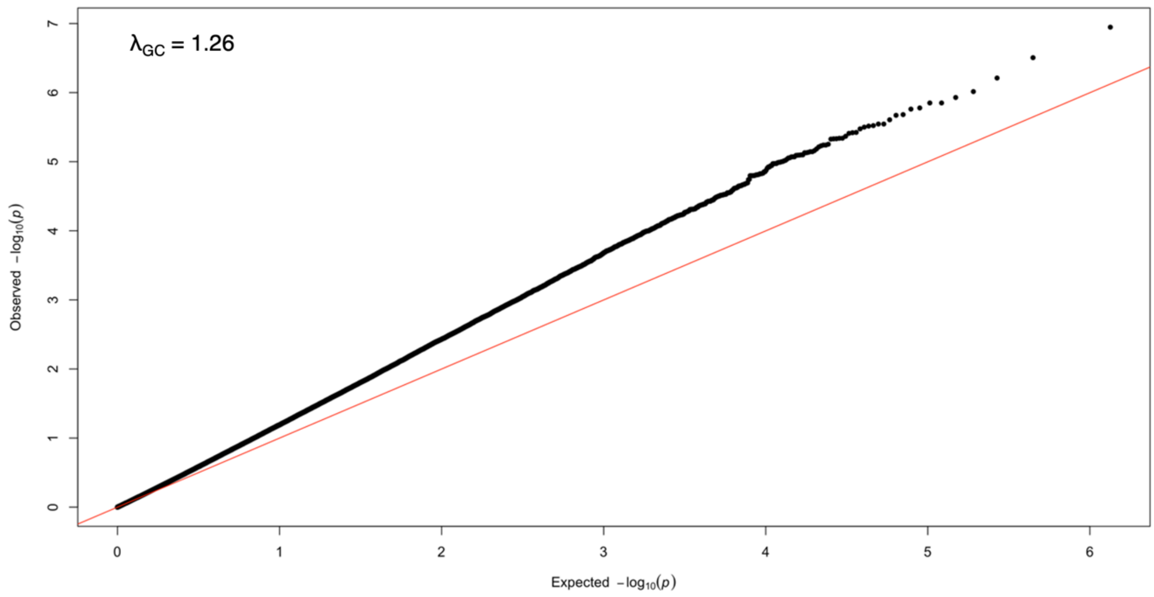

Supplementary Figure 4. **QQ plot showing the observed vs expected SNPs $\log^{10}$ p-values distribution from the BVDV GWAS.**

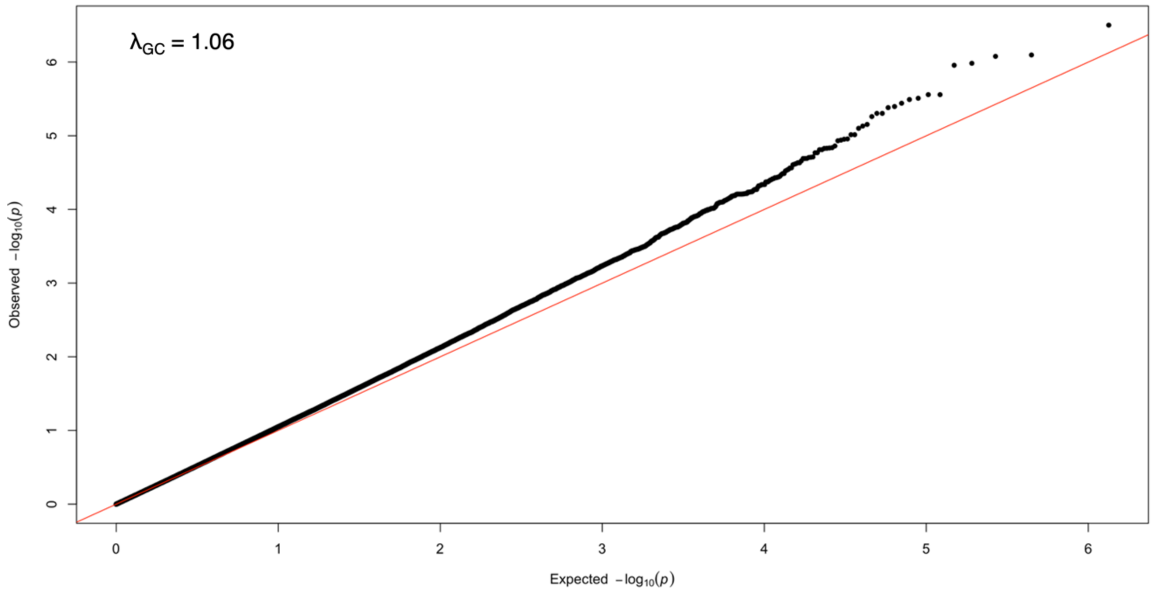

Supplementary Figure 5. **QQ plot showing the observed vs expected SNPs $\log^{10}$ p-values distribution from the *Neospora caninum* GWAS.**

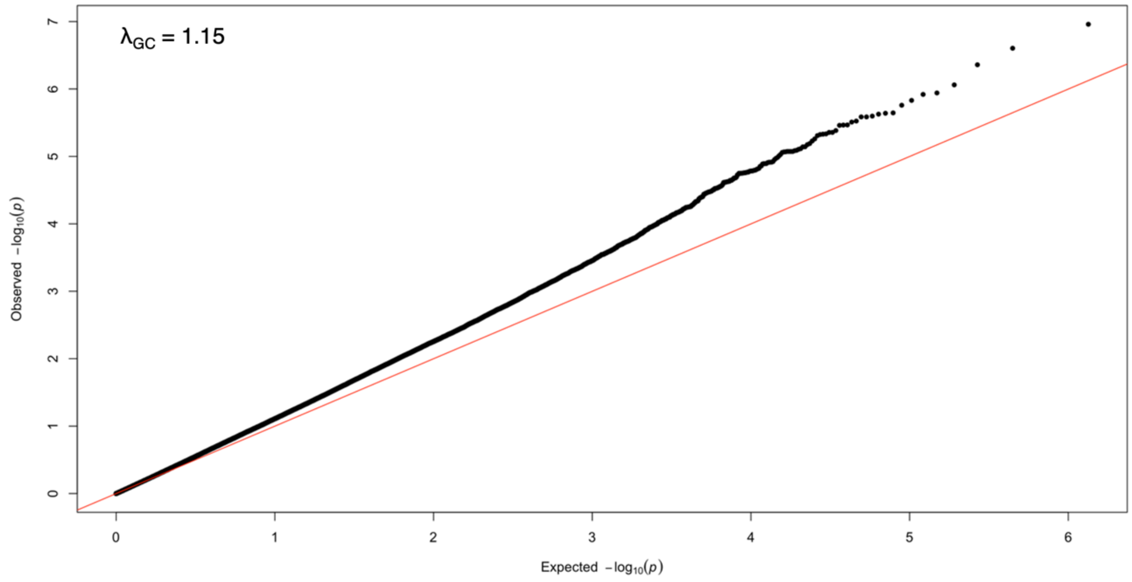

Supplementary Figure 6. **QQ plot showing the observed vs expected SNPs log^{10} p-values distribution from the *Leptospira* hardjo GWAS.**

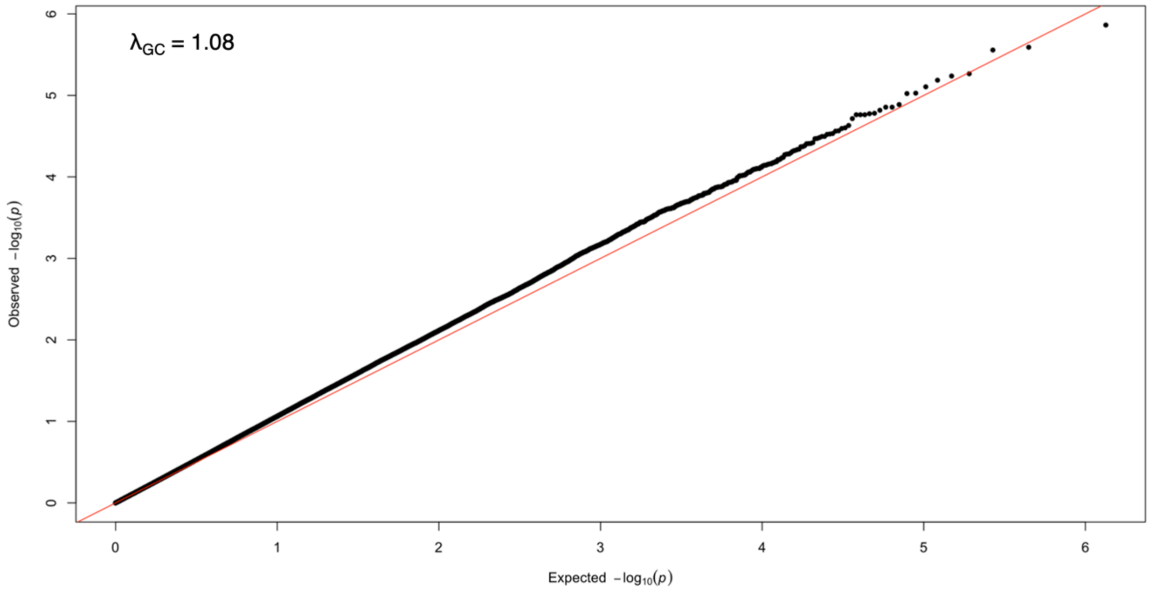

Supplementary Figure 7. **QQ plot showing the observed vs expected SNPs log^{10} p-values distribution from the RVFV GWAS.**

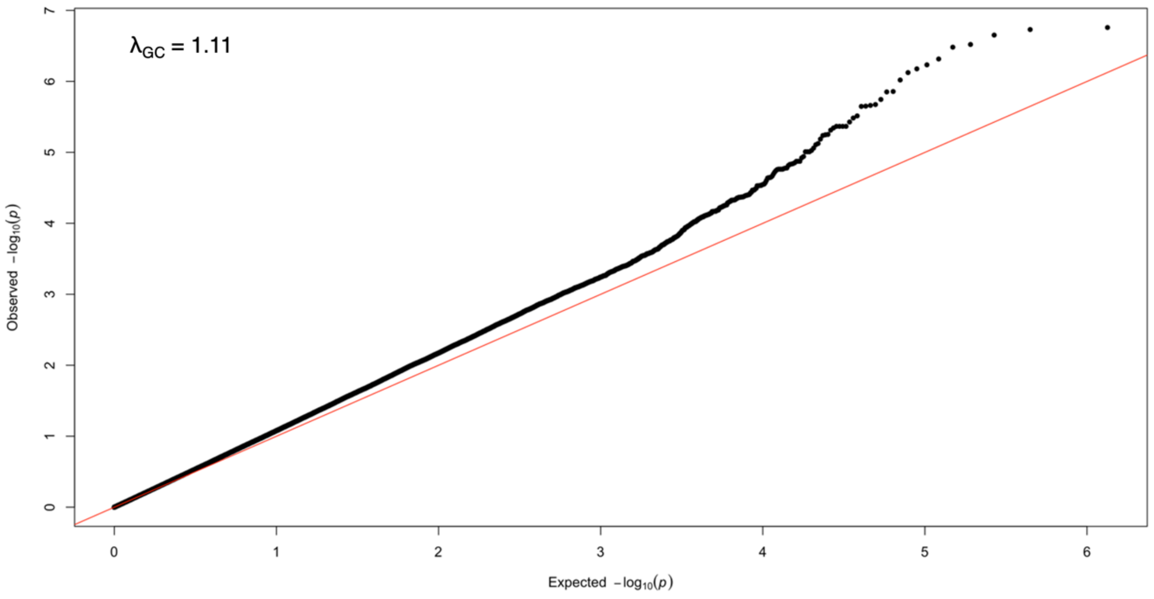

Supplementary Figure 8. **QQ plot showing the observed vs expected SNPs log^{10} p-values distribution from the *Toxoplasma gondii* GWAS.**

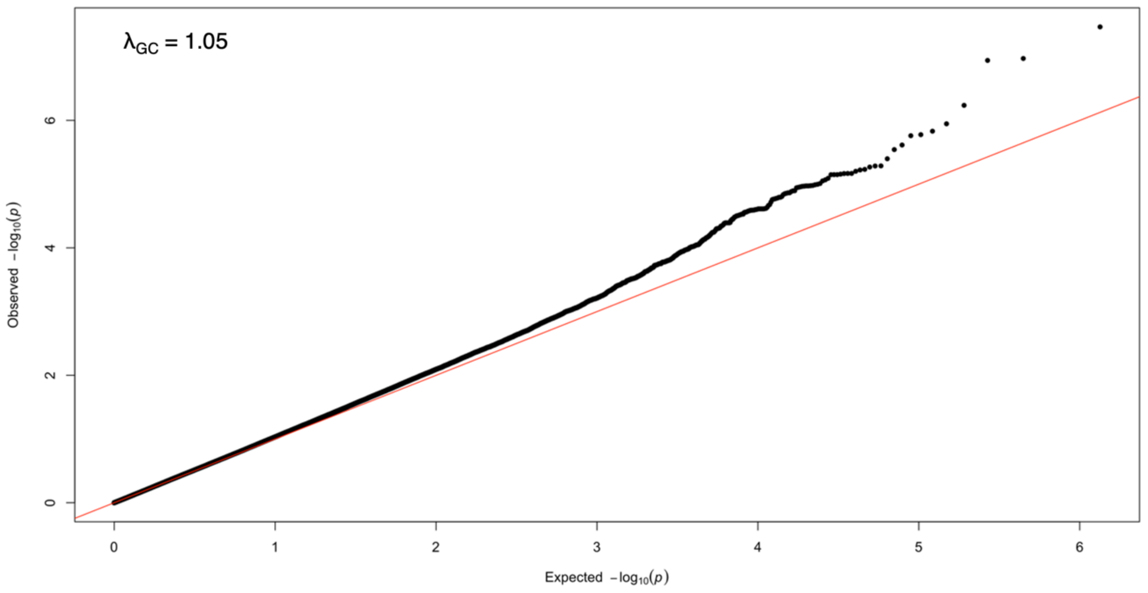

Supplementary Figure 9. **QQ plot showing the observed vs expected SNPs log^{10} p-values distribution from the *Coxiella burnetii* GWAS.**

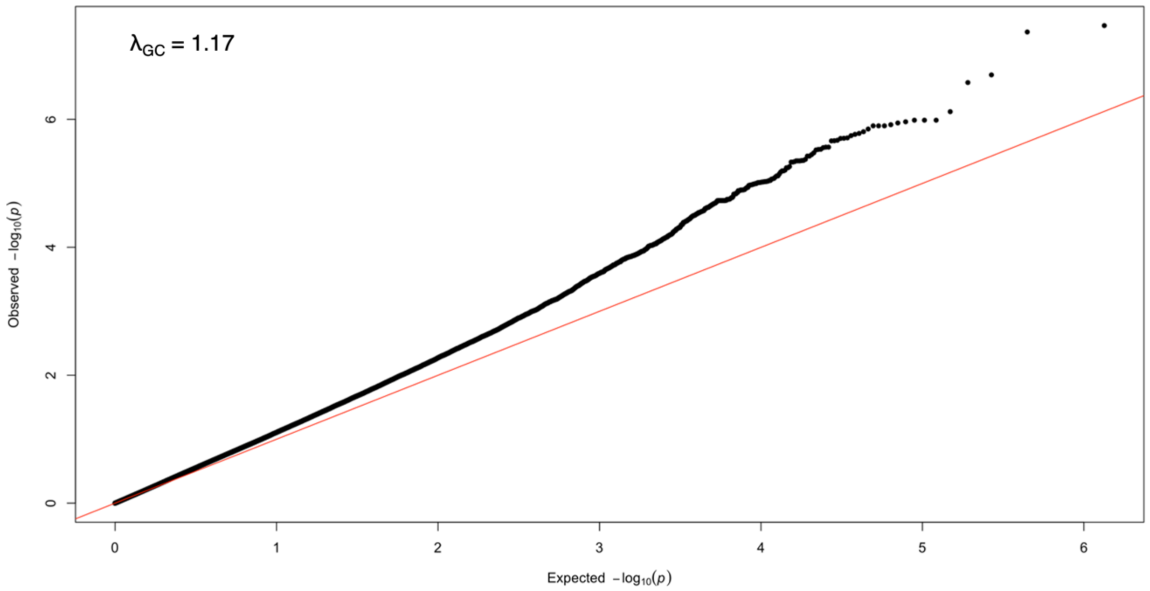

Supplementary Figure 10. **QQ plot showing the observed vs expected SNPs log^{10} p-values distribution from the *Brucella abortus* GWAS.**

1.2 Supplementary Tables

S1 Table. Summary of SNP makers density per chromosome.

Chromosome	Total SNPs
1	42309
2	36329
3	32488
4	31811
5	31458
6	32489
7	30371
8	30511
9	28529
10	27830
11	29330
12	23677
13	21176
14	22353
15	22369

Chromosome	Total SNPs
16	21811
17	20306
18	17472
19	17155
20	19736
21	18915
22	16559
23	13893
24	17128
25	11656
26	13791
27	12314
28	11917
29	13228

S2 Table. Summary statistics of SNP makers showing strong association to serostatus for a given pathogen in GWAS. SNP substitution **(a)** and dominance **(dom)** effects with their standard errors **(SE)** and p-values, proportion of additive genetic **(PvarGen)** and phenotypic **(PvarPhe)** variance and the percentage of genetic variance due to SNP (**% VaSNP**) is provided.

Rs ID	a	p-value (a)	dom	SE (dom)	p-value (dom)	PvarGen	PvarPhe	% VaSNP
**BVDv**								
rs135177308	0.06	1.24×10^{-4}	0.01	0.02	3.56×10^{-1}	0.02	0.01	0.92
rs42731467	-0.08	5.27×10^{2}	0.03	0.04	3.36×10^{-1}	0.03	0.01	1.29
Neospora caninum								
rs43566009	-0.29	1.47×10^{-2}	-0.13	0.12	2.15×10^{-1}	0.05	0.01	1.13
rs43680152	-0.07	3.67×10^{-7}	0.00	0.02	3.85×10^{-1}	0.01	0.00	0.21
rs137229140	-0.30	1.16×10^{-1}	-0.10	0.20	3.49×10^{-1}	0.08	0.02	1.76
rs43059701	-0.09	1.37×10^{-2}	-0.01	0.04	3.92×10^{-1}	0.01	0.00	0.27
***Leptospira* hardjo**								
rs137638789	0.05	3.39×10^{-4}	-0.01	0.02	3.43×10^{-1}	0.04	0.01	1.20
rs109573926	-0.06	1.01×10^{-6}	-0.04	0.01	2.31×10^{-2}	0.04	0.01	1.14
Toxoplasma gondii								
rs42429616	0.01	3.96×10^{-1}	0.14	0.12	1.94×10^{-1}	0.07	0.01	1.33
rs110528913	-0.14	4.85×10^{-2}	-0.02	0.07	3.86×10^{-1}	0.05	0.01	0.88
rs41729510	-0.24	5.84×10^{-3}	-0.15	0.09	9.00×10^{-2}	0.06	0.01	1.20
Coxiella burnetiid								
rs136314037	0.01		0.15	0.03	4.03×10^{-6}	0.25	0.01	1.21
rs132672447	0.01		0.17	0.03	7.82×10^{-8}	0.34	0.02	1.64
rs137170014	-0.08	1.41×10^{-4}	-0.03	0.02	1.22×10^{-1}	0.29	0.01	1.39
Brucella abortus								
rs43008805	-0.09	9.26×10^{-4}	-0.06	0.03	4.04×10^{-2}	0.07	0.01	0.88
